# *ADIPOQ*,*KCNJ11* and *TCF7L2* polymorphisms in type 2 diabetes in Kyrgyz population: A case‐control study

**DOI:** 10.1111/jcmm.14061

**Published:** 2018-11-23

**Authors:** Jainagul Isakova, Elnura Talaibekova, Denis Vinnikov, Iskender Saadanov, Nazira Aldasheva

**Affiliations:** ^1^ Institute of Molecular Biology and Medicine Bishkek Kyrgyzstan; ^2^ Al‐Farabi Kazakh National University, School of Public Health Almaty Kazakhstan; ^3^ Biological Institute National Research Tomsk State University Tomsk Russian Federation

**Keywords:** ADIPOQ, gene, KCNJ11, polymorphism, TCF7L2, type 2 diabetes

## Abstract

The aim of this study was to ascertain the polymorphic markers profile of *ADIPOQ*,*KCNJ11* and *TCF7L2* genes in Kyrgyz population and to analyze the association of polymorphic markers and combinations of *ADIPOQ* gene's G276T locus, *KCNJ11* gene's Glu23Lys locus and *TCF7L2* gene's VS3C>T locus with type two diabetes (T2D) in Kyrgyz population. In this case‐control study, 114 T2D patients 109 non‐diabetic participants were genotyped using polymerase chain reaction‐restriction fragment length polymorphism (PCR‐RFLP). Two individual polymorphisms (*ADIPOQ* rs1501299, *KCNJ11* rs5219) were found to be associated with T2D. We found two (Lys23Lys/CC and Glu23Lys/CT) of the overall nine combinations, which were more prevalent in T2D group compared to controls (χ^2^ = 4.21, P = 0.04). Lys23Lys/CC combination was associated with a 2.65‐fold increased likelihood of T2D (OR = 2.65, 95% CI 1.12‐6.28), whereas the Glu23Lys/CT combination also increased such likelihood (OR = 3.88, 95% CI 1.27‐11.91). This study demonstrated some association of 276T allele and *ADIPOQ* gene G276T heterozygous genotype as well as *KCNJ11* gene 23Lys allele with T2D in ethnic Kyrgyz, but study results should be interpreted with caution because of the limited statistical power.

## 1 INTRODUCTION

Diabetes mellitus type 2 (T2D) is one of the major threats to human health because of its high prevalence, persistently growing prevalence and the severity of its late vascular complications if unrecognized and untreated.^1^ Up to 50% of T2D cases get diagnosis at years 5‐7 of the disease, while specific macro‐ and micro‐lesions already exist in 20%‐30% patients at that time.^1^

Genetic predisposition to T2D was studied widely during the last decades.^2^, ^3^, ^4^ At present, around 50 candidate genes are considered to increase the likelihood of T2D, of which adiponectin gene (*ADIPOQ*), potassium channel, inwardly rectifying subfamily J, member 11 (*KCNJ11*) and transcription factor 7‐like 2 (*TCF7L2* [IVS3C>T]) may be associated with insulin resistance and β‐cells dysfunction.^5^, ^6^, ^7^, ^8^, ^9^, ^10^, ^11^, ^12^, ^13^ Carriage of various SNP combinations may explain clinical heterogeneity of this disease. Therefore, the analysis of cumulative SNP contribution in T2D is important.

Large variability in genetic markers of T2D between population groups necessitates studies of ethnicity in genetic risks quantification. Genetic profile of *ADIPOQ* (c.G276T, rs1501299), *KCNJ11* (g.5635A>G, rs5219) and *TCF7L2* (g.53341C>T, rs7903146) genes in Kyrgyz population, as well as the association with T2D have never been studied before. The aim of this study was to ascertain the polymorphic markers profile of *ADIPOQ*,* KCNJ11* and *TCF7L2* genes in Kyrgyz population and to analyse the association of polymorphic markers and combinations of *ADIPOQ* gene's G276T locus, *KCNJ11* gene's Glu23Lys locus and *TCF7L2* gene's VS3C>T locus with T2D in Kyrgyz population.

## 2 MATERIALS AND METHODS

This case‐control study with the total sample size N = 223 of unrelated participants of Kyrgyz ethnicity comprised 114 patients, including 61 men (53.5%) and 53 women (46.5%) with clinically verified T2D. 109 patients, including 61 (56%) men and 48 (44%) women without T2D, obesity and with normal total cholesterol and glucose were controls. Patients were included in the main group when they had T2D, diagnosed with World Health Organization (WHO, 1999) criteria and the disease duration was at least 1 year since diagnosis. Patients with pregnancy, severe metabolic decompensation and the combination of two metabolic diseases were excluded. Both cases and controls comprised men and women 18 years and older, all of Kyrgyz ethnicity, born on the territory of Kyrgyz Republic and unrelated with each other. The study was approved by the local Institutional Review Board of the Institute of Molecular Biology and Medicine of Kyrgyz Republic (Minutes #11 of January 14, 2016). Biological material and self‐reported demographic data collection complied with ethical requirements and all patients provided informed consent.

DNA was extracted from blood using the standard method of phenol‐chloroform extraction. We used PCR‐RFLP method to genotype polymorphic loci. We used χ^2^ with Yates correction to compare the frequency of alleles and genotypes in the polymorphic markers under study with T2D. The odds ratio (OR) was the measure of association of a given genotype with the outcome for the minor allele of each analysed locus with its corresponding 95% confidence interval (CI). The differences in *P* < 0.05 were considered statistically significant. We also tested the distribution of allele and genotype frequencies in the population under study using Hardy‐Weinberg equilibrium.

## 3 RESULTS

Two individual polymorphisms (*ADIPOQ* rs1501299, *KCNJ11* rs5219) were found to be associated with type 2 diabetes (Table 1). We found statistically significant differences in the distribution of genotypes and alleles of G276T polymorphic marker of *ADIPOQ* gene between cases and controls. T2D patients had statistically significant greater prevalence of G276T heterozygous genotype (χ2 = 6.65; *P* = 0.037) and 276T allele (χ2 = 5.51; *P* = 0.025) when compared to controls. G276T heterozygous genotype was associated with almost twofold higher risk of T2D (OR = 1.8, 95% CI 1.05‐3.05), whereas 276T allele yielded the OR 1.68 (95% CI 1.09‐2.60). Our sample of 114 cases, OR of G276T genotype 1.8, 37% exposure in controls and 1:1 case:control ratio yielded 59% statistical power. Homozygous genotype G276G was protective against T2D (OR = 0.51, 95% CI 0.30‐0.86), as was G allele (OR = 0.59, 95% CI 0.38‐0.92).

**Table 1:** The distribution of alleles and genotypes of *KCNJ11*,* ADIPOQ* and *TCF7L2* genes in cases and controls

Gene/polymorphism (rs)	Genotypes and alleles	T2D cases, n = 114	Controls, n = 109	χ^2^	*P*	OR	95% CI
*ADIPOQ* c.G276T, (rs1501299)	GG	44.7% (51)	61.5% (67)	6.65	0.037	0.51	0.30‐0.87
	GT	50.9% (58)	36.7% (40)			1.80	1.05‐3.05
	TT	4.4% (5)	1.8% (2)			2.45	0.47‐12.93
	GG	44.7% (51)	61.5% (67)	6.26	0.012	0.51	0.30‐0.87
	GT/TT	55.3% (63)	38.5% (42)			1.97	1.16‐3.36
	TT	4.4% (5)	1.8% (2)	1.19	0.446	2.45	0.47‐12.93
	GT/GG	95.6% (109)	98.2% (107)			0.41	0.08‐2.15
	Allele G	70.2%	79.8%	5.51	0.025	0.59	0.38‐0.92
	Allele T	29.8%	20.2%			1.68	1.09‐2.60
*KCNJ11* Glu23Lys, (rs5219)	Lys23Lys	20.2% (23)	13.8% (15)	6.20	0.045	1.58	0.78‐3.23
	Glu23Lys	47.4% (54)	37.6% (41)			1.49	0.87‐2.55
	Glu23Glu	32.5% (37)	48.6% (53)			0.51	0.29‐0.87
	Glu23Glu	32.5% (37)	48.6% (53)	6.05	0.015	0.51	0.29‐0.87
	Glu23Lys/Lys 23Lys	67.5% (77)	51.4% (56)			1.97	1.14‐3.39
	Lys23Lys	20.2% (23)	13.8% (15)	1.62	0.203	1.58	0.78‐3.23
	Glu23Lys/Glu23Glu	79.8% (91)	86.2% (94)			0.63	0.31‐1.29
	Allele Lys	44%	32.6%	6.01	0.019	1.62	1.10‐2.38
	Allele Glu	56%	67.4%			0.62	0.42‐0.91
*TCF7L2* g.53341C>T (rs7903146)	TT	2.6% (3)	3.7% (4)	0.50	0.772	0.71	0.16‐3.25
	CT	17.5% (20)	14.7% (16)			1.24	0.60‐2.53
	CC	79.8% (91)	81.7% (89)			0.89	0.46‐1.73
	CC	79.8% (91)	81.7% (89)	0.12	0.730	0.89	0.46‐1.73
	CT/TT	20.2% (23)	18.3% (20)			1.12	0.58‐2.19
	TT	2.6% (3)	3.7% (4)	0.20	0.717	0.71	0.16‐3.25
	CT/CC	97.4% (111)	96.3% (105)			1.41	0.31‐6.45
	Allele T	11.4%	11.0%	0.02	0.98	1.04	0.58‐1.87
	Allele C	88.6%	89.0%			0.96	0.53‐1.73

*Note*. T2D: diabetes type 2; OR: odds ratio; CI: confidence interval.

With regard to gene *KCNJ11* Glu23Lys polymorphic locus, we found higher 23Lys allele prevalence in T2D group (44%) compared to controls (32.6%, *P* = 0.019). T2D risk was 1.62 times greater in those carrying 23Lys allele compared to Glu23 (OR = 1.62; 95% CI 1.10‐2.38) carriers. Therefore, 23Lys allele of *KCNJ11* gene is the allele of higher T2D risk in Kyrgyz population.

*ADIPOQ* (G276T), *KCNJ11* (Glu23Lys) and *TCF7L2* (IVS3C/T) genes are located on different chromosomes and are not bound. When standardising genotyping data (z‐clasterisation), we found no correlations between the polymorphic loci under study.

We found two‐ and three‐locus gene‐gene interactions of *ADIPOQ*,* KCNJ11* and *TCF7L2*, which were significantly associated with T2D. Three of the total number of nine genotypes were associated with higher risk of T2D with varying effect (Table 2).

**Table 2:** Significant genotype combinations of G276T polymorphic locus of *ADIPOQ* gene*,* Glu23Lys locus of *KCNJ11* gene and IVS3C>T locus of *TCF7L2* gene in predicting the risk of T2D in Kyrgyz population

Genotype combinations	T2D group n = 114 (%)	Controls n = 109 (%)	OR (95% CI)	χ^2^/*P*
*ADIPOQ* (G276T)+*KCNJ11* (Glu23Lys)				
G276T+Glu23Lys	25 (22)	13 (12)	4.88 (1.93‐12.35)	10.36/0.0013
G276G+Lys23Lys	11 (9.6)	6 (5.5)	4.65 (1.42‐15.21)	5.53/0.019
G276T+Glu23Glu	22 (19.3)	18 (16.5)	3.10 (1.27‐7.59)	5.28/0.022
*ADIPOQ* (G276T)+*TCF7L2* (IVS3C>T*)*				
G276T+CC	48 (42)	34 (31)	1.97 (1.07‐3.61)	4.21/0.04
*KCNJ11* (Glu23Lys)+*TCF7L2* (IVS3C>T)				
Lys23Lys+CC	21 (18)	11 (10)	2.65 (1.12‐6.28)	4.13/0.042
Glu23Lys+CT	14 (12)	5 (5)	3.88 (1.27‐11.91)	4.91/0.027
*ADIPOQ* (G276T)+*KCNJ11* (Glu23Lys)+*TCF7L2* (IVS3C>T*)*				
G276T/Glu23Lys /CT	6 (5)	1 (1)	14.48 (1.52‐134.1)	5.60/0.02

*Note*. T2D: diabetes type 2; OR: odds ratio; CI: confidence interval.

Diabetes mellitus type 2 patients had a statistically significant higher prevalence of G276T/CC genotypes combination (42%) compared to controls (31%) (Table 2). G276T/CC combination was associated with a double risk of T2D (OR = 1.97, 95% CI 1.07‐3.61). When taken separately, the OR of T2D was 1.8 in G276T polymorphic marker carriers and 0.89 in those carrying CC of *TCF7L2* gene. We found two (Lys23Lys/CC and Glu23Lys/CT) of the overall nine combinations, which were more prevalent in T2D group compared to controls (χ^2^ = 4.21, *P* = 0.04). Lys23Lys/CC combination was associated with a 2.65‐fold increased likelihood of T2D (OR = 2.65, 95% CI 1.12‐6.28), whereas the Glu23Lys/CT combination also increased such likelihood (OR = 3.88, 95% CI 1.27‐11.91). When taken separately, the OR of T2D was 1.49 in Glu23Lys polymorphic marker carriers and 1.24 in those carrying CT of *TCF7L2* gene.

## 4 DISCUSSION

This study demonstrated some association of 276T allele and *ADIPOQ* gene G276T heterozygous genotype as well as *KCNJ11* gene 23Lys allele with T2D in ethnic Kyrgyz, but study results should be interpreted with caution because of the limited statistical power.

Literature data support the association of *KCNJ11* gene 23Lys allele with T2D in the Chinese, Japanese, Koreans, Russians, British, Tunisian, Taiwanese and Iranians.^5^, ^6^, ^7^ *KCNJ11* gene Glu23Lys polymorphic marker's association with T2D is likely associated with the replacement of glutamate with lysine in the 23rd position of Kir 6.2 protein, which results in suppressed insulin blood secretion as a result of increased ATP‐dependent ionic channel activity, membrane potential change and intracellular calcium concentration decrease, which initiates insulin secretion.^2^, ^5^, ^6^, ^7^ The association of *ADIPOQ* gene G276T polymorphic locus with T2D in our study was also previously confirmed in the Japanese and Taiwanese,^8^, ^9^ and such association may be explained with impaired tissue susceptibility to insulin.^2^, ^8^, ^9^

*TCF7L2* gene IVS3C/T polymorphic locus was not associated with T2D in the current analysis. Asian and Caucasian populations largely differ in the prevalence of IVS3‐T allele of TCF7L2 gene IVS3C/T polymorphism. Asian population studies showed lower IVS3‐T prevalence (5%‐15%) as opposed to Caucasian ones (36%‐46%), and even up to 50% in African populations.^10^ IVS3‐T allele prevalence (11%) in Kyrgyz population was somewhat similar to other Asian populations.^11^ Literature data support the role of IVS3‐T allele of *TCF7L2* gene IVS3C/T polymorphic locus with T2D in the European populations.^10^ In Asian populations, *TCF7L*2 gene IVS3C>T polymorphic locus is either not associated with T2D separately or has a weak association.^11^, ^12^, ^13^

Studies of genetic background of T2D are vital to timely identify high‐risk individuals and to implement a prevention plan in these individuals as well as in general population.

## CONFLICT OF INTEREST

The authors confirm that there are no conflicts of interest.

## References

[1] Zimmet PZ . Diabetes and its drivers: the largest epidemic in human history? Clin Diabetes Endocrinol. 2017;3:1. 28702255 10.1186/s40842-016-0039-3 PMC5471716

[2] Cho YS , Lee J‐Y , Park KS , et al. Genetics of type 2 diabetes in East Asian populations. Curr Diab Rep. 2012;12:686‐696. 22993124 10.1007/s11892-012-0326-z

[3] Qi Q , Hu FB . Genetics of type 2 diabetes in European populations. J Diabetes. 2012;4:203‐212. 22781158 10.1111/j.1753-0407.2012.00224.x PMC3422419

[4] Cook JP , Morris AP . Multi‐ethnic genome‐wide association study identifies novel locus for type 2 diabetes susceptibility. Eur J Hum Genet. 2016;24:1175. 27189021 10.1038/ejhg.2016.17 PMC4947384

[5] Zhou D , Zhang D , Liu Y , et al. The E23K variation in the KCNJ11 gene is associated with type 2 diabetes in Chinese and East Asian population. J Hum Genet. 2009;54:433‐435. 19498446 10.1038/jhg.2009.54

[6] Sakamoto Y , Inoue H , Keshavarz P , et al. SNPs in the KCNJ11‐ABCC8 gene locus are associated with type 2 diabetes and blood pressure levels in the Japanese population. J Hum Genet. 2007;52:781‐793. 17823772 10.1007/s10038-007-0190-x

[7] Koo BK , Cho YM , Park BL , et al. Polymorphisms of KCNJ11 (Kir6. 2 gene) are associated with type 2 diabetes and hypertension in the Korean population. Diabet Med. 2007; 24: 178‐186. 17257281 10.1111/j.1464-5491.2006.02050.x

[8] Hara K , Boutin P , Mori Y , et al. Genetic variation in the gene encoding adiponectin is associated with an increased risk of type 2 diabetes in the Japanese population. Diabetes. 2002;51:536‐540. 11812766 10.2337/diabetes.51.2.536

[9] Tsai M‐K , Wang H‐MD , Shiang J‐C , et al. Sequence variants of ADIPOQ and association with type 2 diabetes mellitus in Taiwan Chinese Han population. Sci World J. 2014;2014:1‐7. 10.1155/2014/650393 PMC4121223 25121131

[10] Cauchi S , Meyre D , Dina C , et al. Transcription factor TCF7L2 genetic study in the French population: expression in human β‐cells and adipose tissue and strong association with type 2 diabetes. Diabetes. 2006;55:2903‐2908. 17003360 10.2337/db06-0474

[11] Horikoshi M , Hara K , Ito C , et al. A genetic variation of the transcription factor 7‐like 2 gene is associated with risk of type 2 diabetes in the Japanese population. Diabetologia. 2007;50:747‐751. 17245589 10.1007/s00125-006-0588-6

[12] Ren Q , Han XY , Wang F , et al. Exon sequencing and association analysis of polymorphisms in TCF7L2 with type 2 diabetes in a Chinese population. Diabetologia. 2008;51:1146‐1152. 18493736 10.1007/s00125-008-1039-3

[13] Chang Y‐C , Chang T‐J , Jiang Y‐D , et al. Association study of the genetic polymorphisms of the transcription factor 7‐like 2 (TCF7L2) gene and type 2 diabetes in the Chinese population. Diabetes. 2007;56:2631‐2637. 17579206 10.2337/db07-0421

